# Dual role of Apolipoprotein D as long-term instructive factor and acute signal conditioning microglial secretory and phagocytic responses

**DOI:** 10.3389/fncel.2023.1112930

**Published:** 2023-01-26

**Authors:** Miriam Corraliza-Gomez, Beatriz Bendito, David Sandonis-Camarero, Jorge Mondejar-Duran, Miguel Villa, Marta Poncela, Jorge Valero, Diego Sanchez, Maria D. Ganfornina

**Affiliations:** ^1^Instituto de Biología y Genética Molecular, Unidad de Excelencia, University of Valladolid-CSIC, Valladolid, Spain; ^2^Instituto de Neurociencias de Castilla y León, University of Salamanca, Salamanca, Spain

**Keywords:** microglia, cytokine secretion, myelin phagocytosis, amyloid-beta endocytosis, membrane-binding protein, immune memory, acute response, astrocyte-microglia crosstalk

## Abstract

Microglial cells are recognized as very dynamic brain cells, screening the environment and sensitive to signals from all other cell types in health and disease. Apolipoprotein D (ApoD), a lipid-binding protein of the Lipocalin family, is required for nervous system optimal function and proper development and maintenance of key neural structures. ApoD has a cell and state-dependent expression in the healthy nervous system, and increases its expression upon aging, damage or neurodegeneration. An extensive overlap exists between processes where ApoD is involved and those where microglia have an active role. However, no study has analyzed the role of ApoD in microglial responses. In this work, we test the hypothesis that ApoD, as an extracellular signal, participates in the intercellular crosstalk sensed by microglia and impacts their responses upon physiological aging or damaging conditions. We find that a significant proportion of ApoD-dependent aging transcriptome are microglia-specific genes, and show that lack of ApoD *in vivo* dysregulates microglial density in mouse hippocampus in an age-dependent manner. Murine BV2 and primary microglia do not express ApoD, but it can be internalized and targeted to lysosomes, where unlike other cell types it is transiently present. Cytokine secretion profiles and myelin phagocytosis reveal that ApoD has both long-term pre-conditioning effects on microglia as well as acute effects on these microglial immune functions, without significant modification of cell survival. ApoD-triggered cytokine signatures are stimuli (paraquat vs. Aβ oligomers) and sex-dependent. Acute exposure to ApoD induces microglia to switch from their resting state to a secretory and less phagocytic phenotype, while long-term absence of ApoD leads to attenuated cytokine induction and increased myelin uptake, supporting a role for ApoD as priming or immune training factor. This knowledge should help to advance our understanding of the complex responses of microglia during aging and neurodegeneration, where signals received along our lifespan are combined with damage-triggered acute signals, conditioning both beneficial roles and limitations of microglial functions.

## Introduction

Apolipoprotein D (ApoD) is a lipid-binding protein of the Lipocalin family known to modulate a wide array of processes, both in health and disease (reviewed by Sanchez and Ganfornina, 2021). Myelinating glia (oligodendrocytes and Schwann cells), mural cells (pericytes), and astrocytes are cell types known to express ApoD in the mammalian nervous system. Adult neurons do not express ApoD but are able to endocytose this protein from the extracellular space, particularly in oxidative stress conditions (Martínez-Pinilla et al., 2015; Pascua-Maestro et al., 2019). ApoD-immunoreactive cells with microglial markers have also been found within compact Aβ plaques in patients with Alzheimer’s disease (Desai et al., 2005).

Functional analyses of ApoD in cellular andx animal models demonstrate that it is required for nervous system optimal function and for the proper development and maintenance of key nervous system structures (reviewed by Sanchez and Ganfornina, 2021). ApoD acts as an endogenous controller of the redox state and composition of cellular lipid structures [e.g., lysosomal or myelin membranes; (Pascua-Maestro et al., 2017; García-Mateo et al., 2018)] and extracellular ones [e.g., HDLs or exosomes; (Sreckovic et al., 2013; Pascua-Maestro et al., 2019)]. This biochemical function has downstream consequences that directly conditions cellular functions (e.g., myelin compaction and lysosome pH, where ApoD has direct effector functions) but can also result in gene expression modulation (where ApoD is expected to have regulator or signaling functions). Transcriptional profiles in response to aging, experimental oxidative stress, or injury have been analyzed in loss-of-function and gain-of-function mutants of mouse ApoD or its Drosophila homologous, revealing ensembles of ApoD-dependent genes (Hull-Thompson et al., 2009; Ganfornina et al., 2010; Bajo-Grañeras et al., 2011a,b; Ruiz et al., 2013; García-Mateo et al., 2014, 2018; Sanchez et al., 2015; Pascua-Maestro et al., 2020).

Apolipoprotein D is well known for its increased expression in the nervous system upon aging, damage or neurodegeneration. Yet, its expression during development or throughout adulthood in the healthy brain is also striking: ApoD is never ubiquitously expressed, never in all cell types in a tissue, or at all times in a given cell type. It is expressed with a salt-and-pepper spatiotemporal pattern, suggesting a fine control that depends on particular physiological states in each cellular niche (Sanchez and Ganfornina, 2021).

These ApoD features, both in a healthy brain or in response to a variety of potentially harmful stimuli, directly connects with one of the most dynamic cells in the brain screening the environment and sensitive to signals from all other cell types: microglial cells (Sierra et al., 2019; Paolicelli et al., 2022). Can ApoD-expressing cells and microglial cells be part of coordinated responses in the brain to different stimuli? If such is the case, it is predicted that pulses of ApoD expression, in the healthy or diseased brain, might be sensed by microglia and influence one or more of the panoply of microglial states and responses.

Microglia, often described as the resident macrophages of the brain, have surprised researchers in the field by having many more functions than expected (Sierra et al., 2019; Paolicelli et al., 2022). In addition to activities set forth in pathological conditions, like release of inflammatory mediators or phagocytosis of cellular debris, they perform surveillance functions, sensing cues in the environment (Davalos et al., 2005; Nimmerjahn et al., 2005), like those derived from neuronal activity or indicators of the functional state of synapses (Wake et al., 2009). They are also key players in the control of neuronal numbers in a circuit, e.g., by determining the final number of Purkinje cells during development, eliminating them by bursts of ROS production (Marín-Teva et al., 2004), or by phagocytosing the surplus of adult newborn neurons (Sierra et al., 2010). Modulation of myelination and remyelination (Healy et al., 2016; Safaiyan et al., 2021), vasculogenesis and blood-brain barrier permeability are processes where microglial cells have also been involved (Tawfik et al., 2014; Haruwaka et al., 2019).

The processes where microglia have an active role and the stimuli to which they respond, have extensive overlap with those in which ApoD is involved in the nervous system: neurotransmission, neuritogenesis and synaptogenesis, functional preservation of neuronal cells upon oxidative stress or Aβ-related challenges, response to tissue damage, by limiting the dimension and duration of gliosis and inflammation, and completion of myelin development, myelin maintenance, and modulation of phagocytic activity of Schwann cells and macrophages (reviewed by Sanchez and Ganfornina, 2021).

In this work, we test the hypothesis that ApoD, as an extracellular signal, is part of the inter-cellular crosstalk that is sensed by microglia and impacts their response in the context of physiological aging and upon damaging conditions (oxidative or amyloid stress). We found an age-dependent response of microglial population in the mouse brain, indicating that ApoD presence *in vivo* does impinge on microglial cells physiology. After characterization of ApoD expression and ApoD traffic within microglial cells, we have studied the two most classical roles of microglia as immune cells of the brain, cytokine secretion profiles and phagocytosis of myelin debris, and found that ApoD has both long-term pre-conditioning effects on microglia, as well as acute effects on both functions of microglia.

## Materials and methods

### Animals, cell cultures, and treatments

C57BL/6J mice were maintained in positive pressure-ventilated racks at 25 ± 1°C with 12 h light/dark cycle, fed *ad libitum* with a standard diet (Global Diet 2014; Harlan Inc., Indianapolis, IN, USA), and allowed free access to filtered and UV-irradiated water. Experimental procedures were approved by the University of Valladolid Animal Care and Use Committee, following the regulations of the Care and Use of Mammals in Research (European Commission Directive 86/609/CEE, Spanish Royal Decree 1201/2005).

The murine microglial BV2 cell line (Blasi et al., 1990) was cultured at 37°C in a humidity-saturated atmosphere containing 5% CO_2_ in RPMI 1640 (Gibco) supplemented with 5 heat-inactivated FBS, 1 L-glutamine, and 1% P/S (100 U/ml penicillin, 100 U/ml streptomycin). The mouse astrocytic IMA2.1 line was cultured in DMEM with 5 FBS, 1 L-glutamine, and 1% P/S.

Mouse primary microglial cells were isolated from individual newborn mice, sexed and cultured as previously described (Corraliza-Gomez, 2021). Briefly, after 21 days in culture of primary mixed glial cells, astrocytes were detached and isolated microglia were re-exposed to the mixed glia-conditioned media during the last 24 h, thus containing astroglial pro-survival and proliferative factors. The conditioned media was supplemented with macrophage colony stimulator factor (M-CSF, 25 ng/ml). Isolated microglia remained for 48–120 h in this media to attain a basal or homeostatic state (Witting and Möller, 2011) before their use in experiments.

Cell treatments with LPS (Sigma-Aldrich), the ROS generator Paraquat (PQ; Sigma-Aldrich) or oligomeric Aβ_1–42_ (Bachem), with or without pre-exposure to ApoD, were performed in serum-free media after a 5–36 h adaptation period. Human ApoD (hApoD) was purified from breast cystic fluid as described (Ruiz et al., 2013). Aβ oligomers were prepared following the protocol by Corraliza-Gomez (2021). Briefly, after solubilization in 1,1,1,3,3,3-Hexafluoropropanol (HFIP; Sigma-Aldrich), aliquots were left for 2 h to evaporate and the peptide film was dissolved in DMSO as a 5 mM stock, which was sonicated and stored at −20°C. Before use, the stock was diluted (1.6 μl/100 μl of DMEM-F12 without additives), sonicated again, and Aβ peptides were allowed to polymerize for 24 h at 4°C (Supplementary Figure 1B). For imaging experiments, fluorescently-labeled Aβ oligomers (FAM-Aβ_1–42_, Bachem) were prepared as described above.

### Immunoblot analysis

Cell lysates, membrane preparations and concentrated supernatants were analyzed by immunoblot under denaturing and reducing conditions (0.5% SDS, 25 mM DTT). Electrophoretically-separated proteins were transferred to PVDF membranes. We used 0.1% SDS transfer buffer for myelin degradation experiments, where membranes were exposed to rabbit serum anti-mouse Mbp (Abcam) and mouse anti-tubulin (Sigma), the latter being used for normalization. Membrane preparation and soluble supernatant transferred-proteins were incubated with rabbit serum anti-hApoD (custom made against purified human ApoD), mouse anti-PMCA (sc-20028; Santa Cruz Biotechnology) and mouse anti-β-amyloid (sc-28365; Santa Cruz Biotechnology). HRP-conjugated secondary antibodies (Santa Cruz Biotechnology) were used as secondary antibodies, and membranes were developed with ECL reagents (Millipore). The ECL signal was recorded with a digital camera (VersaDoc; BioRad), and the band integrated optical density was quantified within the camera linear range to avoid signal saturation.

### Immunohistochemistry, immunocytochemistry, image acquisition, and analysis

Immunohistochemistry on mouse brain paraffin sections was performed as previously described (Sanchez et al., 2015). Immunocytochemistry was carried out in cultured cells attached to poly-L-lysine-treated glass coverslips, fixed with 4% formaldehyde for 10 min at 20°C, and washed in PBS. Fixed cells were blocked and permeabilized with Tween-20 (0.1%) and 1% non-immune (goat or cow) serum. Primary antibodies were rabbit anti-Iba1 (Wako), goat anti-mouse ApoD (Santa Cruz Biotechnology), custom-made rabbit serum anti-hApoD and mouse monoclonal anti-Lamp2 (DSHB). All antibodies were prepared in blocking solution. Secondary antibodies were Alexa Fluor^®^ 594/488-conjugated IgGs (Jackson Labs). After three washes in PBS, cells were mounted in EverBrite™ mounting medium with DAPI and sealed with CoverGrip™ (Biotium).

Immunochemistry fluorescent images were obtained in a Nikon Eclipse80i microscope equipped with a DS-Ri1 digital camera (864 × 614 μm; 3.33 pixels/μm). Images were taken from two sections of the hippocampal region (Supplementary Figure 1A) separated 50 μm, from 3 to 4 mice/age/genotype. Iba1-positive microglial cells were counted with the FIJI program by an independent observer, blind to mouse age and genotype. Immunocytochemistry fluorescence levels were measured with FIJI. Confocal images were obtained with a DMI 6000B microscope/TCS SP5 confocal system (Leica) equipped with AOBS and AOTF systems, a WLL laser, and a 405 line controlled by the LAS AF software (Leica). Details on confocal image acquisition and co-localization analysis with FIJI have been previously reported (Pascua-Maestro et al., 2017).

### RT-PCR

Total RNA from cells or tissue was purified with QIAzol (Qiagen), and its concentration was measured with a Nanodrop spectrophotometer. RNA quality was evaluated by agarose electrophoresis. We reverse-transcribed (PrimeScript, Takara Bio Inc.) 0.5 μg of total RNA with Oligo-dT primers and random hexamers. The resulting reaction was used as a template for standard RT-PCR [cycling conditions: 40x (95°C, 30 s; 60°C, 30 s; 72°C, 30 s)]. Primers used for cDNA amplifications are mouse ApoD-F (5′-CCACCCCAGTTAACCTCACA), ApoD-R (5′-CCACTGTTTCTGGAGGGAGA), mouse RPL18-F (5′-CCATCATGGGAGTGGACAT), and RPL18-R (5′-CACGGCC GTCTTGTTTTC).

### Cytokine quantitation

Primary microglia from two individual mice per sex and genotype were seeded in six well plates, and each well assigned one experimental condition. After 18 h of treatment, conditioned media was centrifuged (1000 g, 10 min, 4°C) to pellet cell debris, and analyzed in 2–3 technical replicas with the bead-based multiplex Magnetic Luminex^®^ Assay (R&D Systems), following the manufacturer protocols. The assay was configured to measure to measure IL1β, TNFα, IL6, IL4, and IL10.

### Myelin isolation, labeling, and phagocytosis assay

Myelin was isolated from 3 month-old mouse brains following a sucrose density-gradient centrifugation method (Norton and Poduslo, 1973) and purified myelin (1 mg/ml) was fluorescently labeled with 12.5 mg/ml of the lipophilic dye 1,1′′-dioctadecyl-3,3,3′,3′-tetramethylindocarbocyanide perchlorate (DiI; Sigma). The details of this procedure have been previously described by García-Mateo et al. (2014) DiI-labeled myelin was stored in small aliquots at −20°C in the dark.

Microglial BV2 cells were incubated with DiI-labeled myelin (25 μg/ml) at 37°C for up to 60 min (Figures 5A, B), followed by washes in PBS to remove unbound myelin. The phagocytic uptake of myelin was studied either by detaching cells followed by flow cytometry (see below), or by fixing cells to the substrate, mounting the coverslips as for immunocytochemistry experiments, and performing a semiautomatic quantification of DiI-labeled particles. To carry out the latter strategy, we recorded the three optical channels of four randomly chosen pictures of 20x microscopy fields for each experimental condition in three independent phagocytosis experiments, maintaining exposure parameters. The DAPI-channel helped us to evaluate cell numbers and their viability state. The TRITC-channel captures the DiI myelin signal. The differential interference contrast (DIC) channel was used to automatically demarcate cell boundaries using the FogBank segmentation method (Chalfoun et al., 2014). Non-overlapping cells were manually selected to define regions of interest (ROIs) and superimpose the segmented boundaries and DiI-labeled phagocytosed particles using a custom-made FIJI macro (Supplementary Figure 3A). This procedure calculated four different variables: (1) number of particles/cell, (2) average area of myelin particles/cell, (3) integrated DiI fluorescence signal/cell, and (4) percent cell area occupied by myelin particles.

**FIGURE 1:**
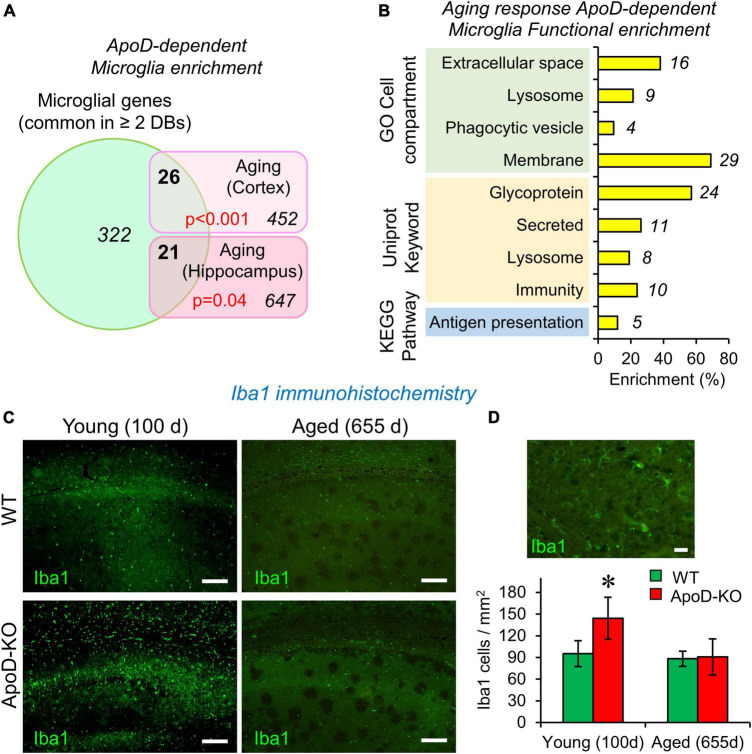
The expression of Apolipoprotein D (ApoD) alters the microglial response to aging. **(A,B)** Bioinformatics analysis of microglia-specific gene enrichment within sets dependent on ApoD expression. Microglia-specific genes are enriched in the set of genes that depend on ApoD for their response to aging in the brain. *P*-values are calculated with a modified Fisher’s exact test **(A)**. Functional enrichment analysis of the set of genes identified in **(A)**, carried out with the DAVID platform **(B)**. **(C)** Young adult mice lacking ApoD expression increase their number of microglial cells in the hippocampus, a genotype-dependent response that disappears upon physiological aging. Degenerated regions in brain tissue are evident in aged samples. Representative images are shown. **(D)** Microglial cells labeled by Iba1 antibody and graphical quantification of Iba1-positive cells in young (100 days) (two serial sections; *n* = 3/genotype) and aged (655 days) (two serial sections; *n* = 4/genotype) samples. Asterisk points to significant differences between young and aged groups (Student’s *t*-test). Calibration bars: 100 μm **(C)**; 20 μm **(D)**.

**FIGURE 2:**
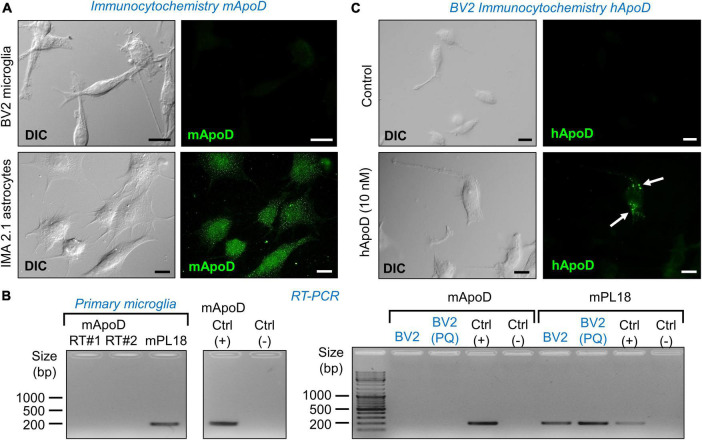
Microglial cells do not express ApoD but are able to incorporate exogenous ApoD in intracellular compartments. **(A)** The mouse microglial cell line BV2 is not labeled by a mouse ApoD antibody, while the astrocytic cell line IMA2.1 shows a characteristic ApoD punctate pattern. **(B)** RT-PCR reveals the absence of ApoD mRNA transcripts in both primary mouse microglia and BV2. PQ 75 μM for 3 h does not induce ApoD expression in BV2 cells. The gene rpl18 was used as a normalizer. RT#1&2 are two separate RT reactions from independent primary cultures. Mouse sciatic nerve expression is used as positive control. An unrelated template is used as negative control. **(C)** Exogenously added human ApoD (hApoD; 10 nM) is internalized to intracellular compartments of BV2 cells after 3 h exposure. Calibration bars: 20 μm.

**FIGURE 3:**
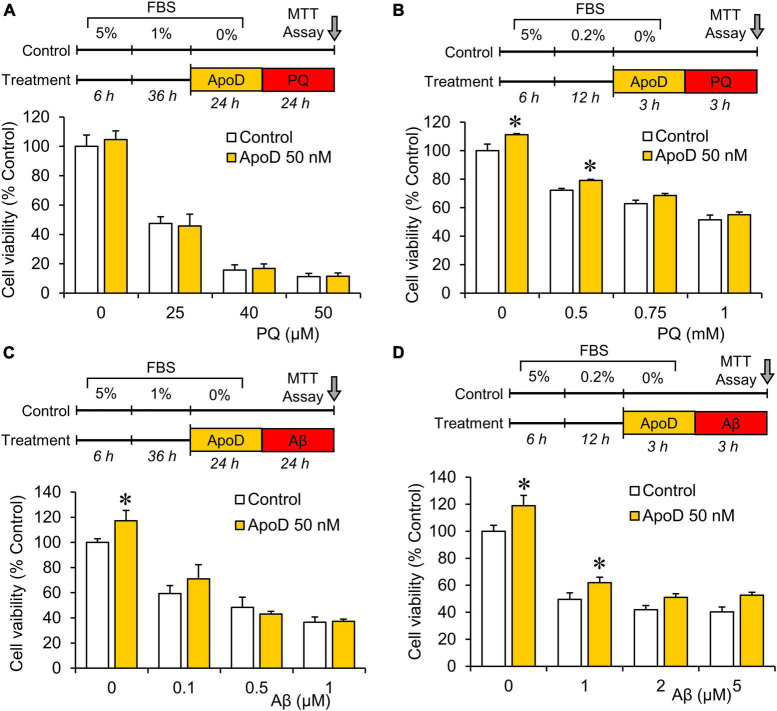
Pre-exposure to purified human ApoD does not rescue BV2 microglial cells from PQ or Aβ-induced cell death. Cell viability analysis of BV2 cells by MTT assay upon PQ **(A,B)** or Aβ **(C,D)** oligomers exposure with or without pre-exposure to ApoD. Treatment started after adaptation of cells to serum starvation (5 - 1 - 0% for 24 h experiments; 5 - 0.2 - 0% for 3 h experiments). Addition of ApoD (50 nM) to the culture medium exerts a mild pro-survival effect on BV2 cells in control conditions but is not sustained with increasing doses of PQ or Aβ. All experiments were performed in triplicates. Asterisks point to significant differences in comparison with control conditions evaluated by ANOVA followed by Holm-Sidak pairwise comparisons.

**FIGURE 4:**
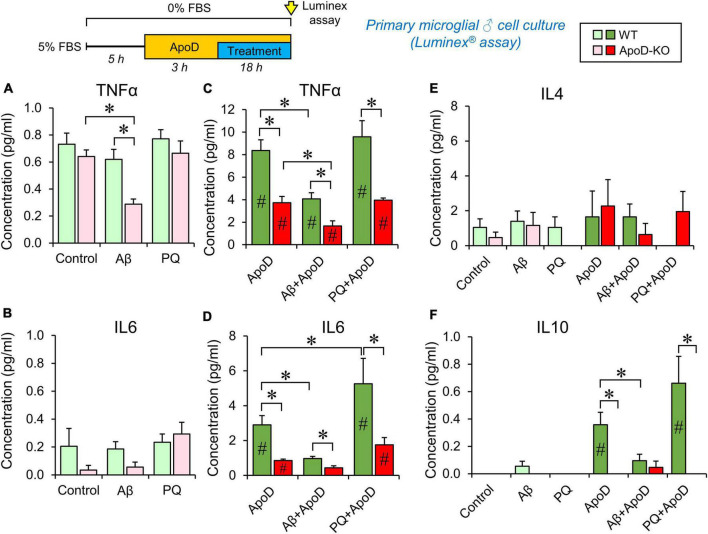
Cytokine secretion profiles of male primary microglia in control conditions, PQ-induced OS or exposure to Aβ-oligomers. Luminex multiplex assay is used on primary microglia culture media after 18 h incubation under different conditions (control, 1 μM Aβ oligomers, or 25 μM PQ). Concentration of each cytokine secreted by WT microglia (shades of green color), and ApoD-KO microglia (shades of red colors) are represented. Light colors (light green or pink) represent values obtained without exposure to ApoD, and dark colors (dark green or red) represent values obtained when stimuli were preceded by the addition of purified human ApoD (50 nM) to the culture medium. ApoD is maintained during the treatment period. Each profile represents 2–3 technical replicas of conditioned media produced by two independent primary cultures/genotype/sex. Bars represent average ± SEM. Asterisks point to significant differences among groups within each graph, and # point to differences between conditions, with or without ApoD acute exposure, evaluated by ANOVA followed by Holm-Sidak pairwise comparisons.

**FIGURE 5:**
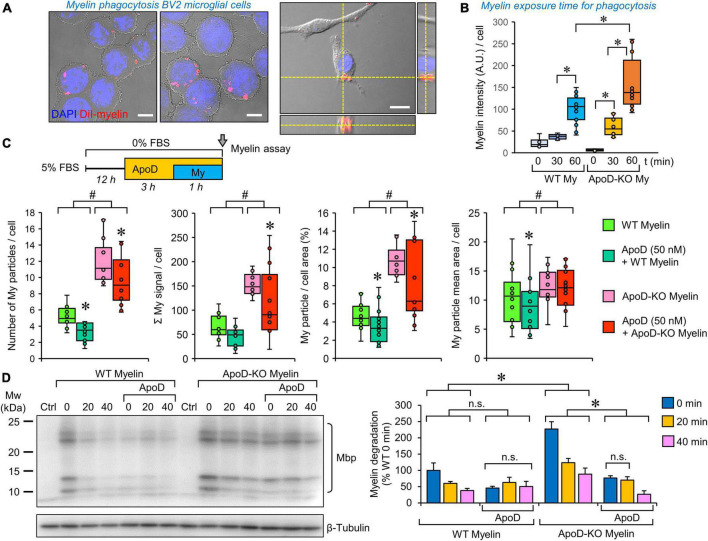
Microglial myelin phagocytosis and degradation depends both on myelin ApoD genotype and exogenous ApoD addition. **(A)** Representative images of differential interference contrast (DIC), DAPI and DiI fluorescence showing that BV2 microglial cells are able to phagocytose DiI-labeled myelin. The right panel shows an orthogonal view derived from a Z-stack to demonstrate the internalized DiI-labeled particles. **(B)** Temporal pattern of DiI-labeled particle incorporation into BV2 cells exposed to WT or ApoD-KO myelin extracts. Total DiI intensity/number of cells is measured (*N* = 3 independent experiments, 6–16 pictures/time point/genotype). Based on these experiments we selected a 60 min incubation time for further analysis. **(C)** Myelin genotype affects the number of phagocytosed myelin particles, their total intensity, the percent area occupied by myelin within the cell, and the mean particle area after 60 min exposure of BV2 microglia to DiI-labeled myelin. A total 398–413 individual cells were measured (*N* = 3 independent experiments, 4 pictures/experimental condition). Each dot in box plots represents the average value/picture. ApoD-KO myelin shows increased values in the four variables analyzed. The addition of exogenous ApoD (50 nM) 3 h before exposure to myelin reduces these values in an ApoD genotype-dependent manner. **(D)** Myelin degradation was assayed by immunoblot against Mbp normalized to β-tubulin (*N* = 3 independent experiments). Four major bands specifically detected by the anti-Mbp antibody were quantified and added to measure the time-course of MBP degradation. Pre-treatment of BV2 cells with exogenous ApoD slows down myelin degradation independently of myelin ApoD genotype. Statistically significant differences were evaluated with ANOVA (two-way in panels **B,C**; three-way in panel **D**) followed by Holm-Sidak pairwise comparisons. # indicate differences due to myelin ApoD genotype (*p* < 0.001). Asterisks indicate differences due to ApoD pre-exposure within each myelin type (*p* < 0.05). Calibration bars: 10 μm (**A**, left panels); 20 μm (**A**, right panel).

### Aβ and myelin phagocytosis quantitation by flow cytometry

Microglial BV2 or primary cells were detached from the culture plates and the fluorescence of FAM-Aβ or DiI-labeled myelin was measured in a Gallios Flow Cytometer (Beckman Coulter). The 575 BP30 channel (560–590 nm) was used to detect labeled myelin, and the 525 BP40 channel (505–545 nm) to detect labeled Aβ, both upon illumination with a 488 nm laser. Fluorescence data were processed with Kaluza Analysis software v.1.3 (Beckman Coulter). We used control histograms of cells not exposed to myelin or exposed to the same carrier concentration used for Aβ (DMSO) to threshold the optimal fluorescence level. A measure of the DiI-labeled myelin or Aβ uptake by microglial cells was estimated by the percent fluorescent cells.

### Crude membrane preparation and *in vitro* membrane-binding analysis

BV2 cell pellets collected in TNE (Tris 50 mM pH 7.4, NaCl 150 mM, EDTA 5 mM) with protease inhibitor cocktail (PI; Roche) were grinded in a Potter glass homogenizer on ice and centrifuged (3,000 g, 10 min). Supernatants were ultracentrifuged (Beckman Optimal-100XP; 100 Ti rotor; 100,000 g, 75 min) and membrane pellets were resuspended in TNE + PI. Protein concentration was quantified with Micro-BCA (Pierce).

The isolated membranes were bath-sonicated (3 min) and incubated with purified hApoD (10–100 nM) for 30 min (20°C, 700 rpm; Eppendorf Thermomixer). Membrane and supernatant fractions were separated again by ultracentrifugation, supernatants were concentrated, and equivalent volumes were analyzed by immunoblot.

### Viability assays

Two methods were used to evaluate cell viability. The MTT assay was performed in adherent cells as previously described (Pascua-Maestro et al., 2018). Briefly, cells were cultured with 3-(4,5-dimethylthiazol-2-yl)-2,5-diphenyltetrazolium bromide (MTT; 62.5 μg/ml) in serum and phenol red-free media for 3 h. Formazan produced by live cells was dissolved by adding an equal volume of an isopropanol solution (isopropanol, 10% Triton X-100). Formazan signal was measured spectrophotometrically (SOFTmax Pro microplate reader; Molecular Devices) at λ = 570 nm after subtracting the λ = 690 nm background.

The LIVE/DEAD^®^ Fixable Near-IR Dead Cell Stain Kit (Invitrogen), based on a fluorescent dye (ex/em 633/755 nm) reacting against cellular amines was used in flow cytometry experiments following the manufacturer specifications. The control sample was divided in two, one of the fractions boiled (99°C) for 5 min, and then combined again to detect the minimum and maximum fluorescence values (live and dead cells, respectively) in the same population.

### Statistical analysis

Statistical analyses were performed with SigmaPlot v.11.0 (Systat) or SPSS v.19 (IBM) software. A *p*-value < 0.05 was used as a threshold for significant changes. The tests used are stated in figure legends.

## Results

### Microglia-specific genes appear significantly enriched in the ApoD-dependent transcriptional profile of brain regions upon physiological aging

The function of ApoD in mammalian nervous system has been intensely studied in neurons, astrocytes and myelinating glia (Sanchez and Ganfornina, 2021). However, no functional link has been established between ApoD and the physiology of microglial cells. We started this research program by performing a gene-enrichment bioinformatic analysis using the lists of mouse brain genes whose transcriptional expression is dependent on ApoD in response to physiological aging in cortex and hippocampus (Sanchez et al., 2015). To perform this analysis (Supplementary File 1), we built a list of microglia-specific genes by comparing three databases of recent RNAseq analyses of microglial cells individually isolated from adult mice (Saunders et al., 2018; Hammond et al., 2019; Jao and Ciernia, 2021). A total of 322 genes were considered specifically expressed by microglia as they are common in ≥ 2 databases. In order to statistically compare the cited lists, an estimate of the number of genes expressed by mouse microglia (15,661 genes) was obtained by averaging the genes reported by Gerrits et al. (2020) and Heng et al. (2021). A modified Fisher’s exact test [EASE score; (Hosack et al., 2003)] was used to estimate microglial enrichment in our gene lists, and revealed that microglial genes are enriched in the set of ApoD-dependent aging-responsive genes in cortex and hippocampus, 26 out of 452 in the cortex and 21 out of 647 in the hippocampus (Figure 1A). The combination of ApoD-dependent microglial genes (42 in total) was subjected to a functional enrichment analysis (fold enrichment ≥ 2; *p*-value EASE < 0.05) using the DAVID platform (Sherman et al., 2022), which uncovered that these genes are enriched in glycoproteins, in proteins secreted or related to membranes and lysosomes, and in those involved in immunity and antigen presentation (Figure 1B). This is an interesting result that blindly identifies functions and subcellular locations now known to be associated with ApoD (Pascua-Maestro et al., 2017, 2019; García-Mateo et al., 2018; Corraliza-Gomez et al., 2022). In summary, these results suggest that the transcriptional response of the aging nervous system to a constitutive absence of ApoD expression is enriched in microglial genes and support the study of a functional relationship of microglia and ApoD.

### Hippocampal microglia density is dysregulated in response to the lack of ApoD in an age-dependent manner

Microglial cell number is known to be tightly regulated throughout adulthood, maintaining a constant value by equilibrium between cell death and cell division (Askew et al., 2017). We evaluated microglial numbers in the hippocampus of young (100 days) and aged (655 days) WT and ApoD-KO mice using the marker Iba1 and found a statistically significant increase of microglial cell density in ApoD-KO young mice (Figures 1C, D). This result indicates the existence of a time window in which microglia are sensing the lack of ApoD and modifying their equilibrium. This alteration early in life is consistent with the fact that alterations conditioning brain aging and degeneration are detected early in the brain of ApoD-KO mice (Sanchez et al., 2015), some of which gets exacerbated with aging while others equalized later in life. This observation further supports the need to pursue an experimental study of the role of ApoD in microglial functions.

### Microglia do not express ApoD, but are able to incorporate exogenous ApoD in intracellular compartments

Since we know that macrophages do not express ApoD (García-Mateo et al., 2014), it is relevant to test whether microglial cells, fulfilling similar functions in the CNS, express ApoD. Fluorescence immunocytochemistry experiments with an anti-mouse ApoD-specific antibody already validated in our laboratory (Pascua-Maestro et al., 2020), demonstrate that the BV2 microglial cells do not have detectable ApoD protein levels, in comparison with a mouse astrocyte cell line (Figure 2A). The same conclusion is reached when measuring the levels of ApoD mRNA by RT-PCR in mouse primary microglia (Figure 2B left) and in the BV2 cell line (Figure 2B right). Exposure of BV2 cells to experimental oxidative stress (OS) with the ROS generator Paraquat (PQ, 75 μM, 3 h) does not induce ApoD mRNA expression either (Figure 2B).

It is well established that ApoD, purified from breast cystic fluid, is internalized by astrocytes and neurons (Do Carmo et al., 2007; Bajo-Grañeras et al., 2011a; Pascua-Maestro et al., 2017, 2019; Corraliza-Gomez et al., 2022). We thus tested whether the exogenous addition of ApoD results in protein internalization in BV2 cells. Immunocytochemistry experiments with a human ApoD-specific antibody confirm this incorporation to intracellular organelles after 3 h exposure to 10 nM hApoD (Figure 2C).

### Pre-exposure to ApoD does not rescue BV2 microglial cells from paraquat or Aβ-induced cell death

Apolipoprotein D has been reported to protect astrocytes, neurons, and fibroblasts when exposed to experimental OS (Bajo-Grañeras et al., 2011a; Pascua-Maestro et al., 2017, 2019, 2020). To test whether ApoD exerts a similar function on microglial cells, we pre-exposed BV2 microglial cells to ApoD at concentrations showing protective effects in astrocytes, before treating them with two stimuli relevant to aging and neurodegeneration (OS and Aβ oligomers). Neither 24 nor 3 h of ApoD pre-exposures rescue microglia significantly from the cell death provoked by PQ or Aβ oligomers (Figure 3). A small pro-survival effect was detected in control conditions or with low dose stimuli.

### ApoD modifies the cytokine response of microglia after oxidative and amyloid stress: Long-term vs. acute effects

We then tested whether ApoD is able to change the profile of cytokines secreted by microglia in response to stressful stimuli (OS and Aβ oligomers), at concentrations that do not compromise viability in primary microglial cells (Supplementary Figure 1C). We used primary microglial cultures obtained from WT or ApoD-KO mice for these experiments, since microglial populations in the mouse brain respond to the life-long absence of ApoD (Figures 1C, D). They are a simplified model of long-term exposure/absence to astrocyte-derived ApoD. We then introduced an acute exposure to ApoD in order to model a damaging event in the nervous system, known to induce acute peaks of ApoD production (Ganfornina et al., 2008). We measured the concentration of five cytokines (IL1β, TNFα, IL6, IL4, and IL10), classically associated with either pro- or anti-inflammatory phenotypes of immune system-related cells. Primary cortical microglia were cultured from either male or female newborn mice of both genotypes, following a protocol with long periods of adaptation to let cells recover from phenotypic changes due to the extraction/culture procedure, and to allow them to reach the basal or ground state of a healthy brain. Multiplex analysis was carried out on media after 18 h culture (in control or experimental conditions) preceded or not by an acute exposure to ApoD. The concentrations of cytokines secreted to the culture media are shown in Figure 4 and Supplementary Figure 2.

Our first finding is that WT microglia, without an acute exposure to ApoD maintain the same profile in control, Aβ or PQ conditions, for TNFα, IL6, and IL4, while IL1β and IL10 secretion appear undetected (Figures 4A, B, E, F; pale green bars). However, ApoD-KO microglia turn down the secretion of TNFα in response to Aβ oligomers specifically (Figure 4A; pink bars). Lower values of IL6 secreted by ApoD-KO microglia are also observed in control and Aβ conditions, though the differences are not statistically significant possibly due to the low quantity of this cytokine, close to the detection threshold of the assay.

Analysis of the responses after acute exposure to ApoD reveals an intriguing result: a significant increase (over 10-fold) of TNFα, IL6, and IL10 secretion (Figures 4C, D, F) is observed, while ApoD pre-treatment does not change IL4 (Figure 4E) or IL1β secretion, still undetected. Moreover, the responses are stimulus-specific, with a significantly less potent inductions of TNFα and IL6 in response to Aβ, and an exacerbated induction of IL6 upon PQ insult (with a similar trend in IL10 response) (Figures 4C, D, F).

The sex of microglial cells does influence their response (Supplementary Figure 2). Female microglia show response patterns for TNFα and IL6 that are globally similar to that of WT or ApoD-KO male microglia. However, they differ from male microglia in: (a) they do not secrete detectable levels of IL10 and IL1β in any of the conditions explored, (b) they show an overall higher level of IL4 secretion (∼fivefold difference with males, Supplementary Figure 2C vs. Figure 4E), and (c) they have a stronger differential TNFα, IL6, and IL4 response to PQ.

An interesting finding in these experiments is the absence of significant amounts of the pro-inflammatory cytokine IL1β in response to any of the experimental conditions tested. In order to check the normal cytokine induction of our primary microglial cultures, we used a classical stimulus (LPS, 100 ng/ml) in WT male microglia (Supplementary Figure 1D). LPS treatment resulted in strong inductions of TNFα, IL6, IL1β, and IL10, while it does not significantly induce IL4 secretion. Pre-exposure to exogenously added purified ApoD did not change the cytokine profile in response to LPS, excepting a significant lower induction of IL6.

### Myelin phagocytosis and degradation by microglia depend both on the myelin ApoD genotype and the exogenous addition of ApoD

To test whether the phagocytic activity of microglia was modulated by ApoD, we performed DiI-labeled myelin phagocytosis assays on BV2 cells, as previously described for peritoneal macrophages (García-Mateo et al., 2014). Exposing cells to myelin isolated from WT or ApoD-KO brains allows us to evaluate if the absence of ApoD in myelin membranes, or its long-term consequences in myelin compaction and structure (García-Mateo et al., 2018), conditions the functional performance of microglial cells. By combining this approach with ApoD pre-exposure, we model a microglia-myelin encounter after an acute ApoD peak. We first selected the engulfment of DiI-myelin by microglial cells after 60 min of incubation (Figure 5A). Quantification of internalized myelin by fluorescence microscopy (Figure 5B) and flow cytometry analysis (Supplementary Figure 3B) demonstrate an increased phagocytic uptake when cells are exposed to myelin obtained from ApoD-KO brains.

To further characterize this effect, a semiautomatic quantification of DiI-labeled particles in substrate-bound BV2 cells (see “Section Materials and methods” and Supplementary Figure 3A) was performed. The data (Figure 5C) indicate that phagocytosed ApoD-KO myelin particles are more numerous, thus occupying more cellular area and increasing the overall DiI intensity labeling per cell, than those of myelin derived from WT mice. Also, even though the starting myelin particle size, analyzed *in vitro*, does not differ with genotype (García-Mateo et al., 2014), a small increase in the mean area of particles is observed for ApoD-KO myelin once incorporated into microglial cells (Figure 5C, far right).

A very interesting result was observed when BV2 cells were acutely exposed to purified ApoD before adding myelin particles to the culture. An overall decrease in all variables characterizing myelin phagocytic uptake is revealed, consistent with the opposite effect observed when BV2 cells were cultured with ApoD-KO myelin (Figure 5C). In contrast, no effect of ApoD exposure on Aβ oligomers uptake was evidenced in primary microglial cells (Supplementary Figure 3C), revealing that different phagocytic phenotypes have a differential sensitivity to ApoD.

Finally, we assayed the degradation of myelin particles incorporated by BV2 cells by immunoblot analysis of cell protein extracts with a Mbp-specific antibody after normalization with β-tubulin (Figure 5D). We evaluated the amounts of Mbp present 20 and 40 min after myelin was removed from the culture media. This approach confirms the increased ApoD-KO myelin phagocytosed during the 60 min exposure to myelin, and shows that myelin degradation progresses at a similar speed regardless the myelin genotype. A decreased incorporation of myelin to BV2 pre-exposed to purified ApoD was also confirmed in these experiments. After acute exposure to ApoD, a lower degradation rate is revealed, that is unrelated to the myelin genotype.

### ApoD traffic in microglia differs from that of other cell types, with less prominent and transient endolysosomal location

The influence of both long-term and acute exposure of microglial cells to ApoD combined with relevant stimuli for aging, neurodegeneration or demyelinating diseases, reveal clear differences with ApoD roles in astrocytes, Schwann cells, neurons, and other cell types previously explored. We therefore investigated if ApoD-membrane interaction, an internalization modulated by OS and a stable localization in the endosome-lysosomal compartment, do take place in microglia.

**FIGURE 6:**
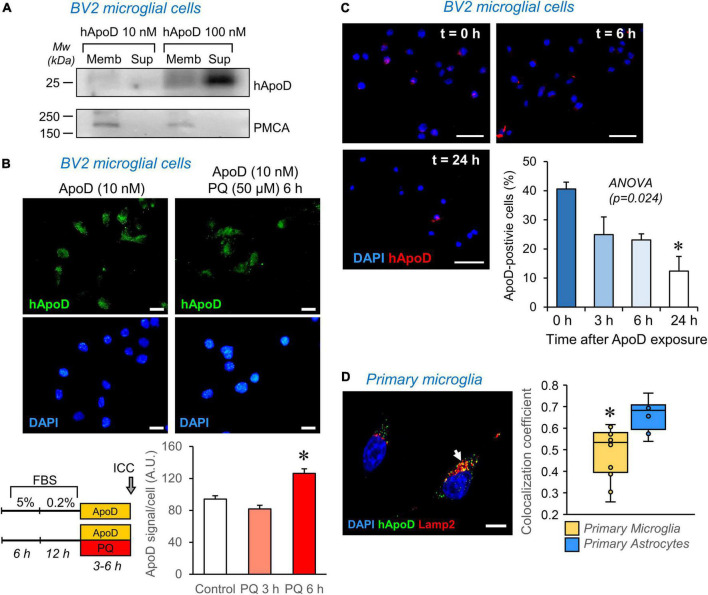
ApoD interacts with microglial cell membranes and is internalized to the late-endosome-lysosomal compartment. **(A)** Human ApoD interacts with BV2 cell membranes in a dose dependent manner. Equivalent volumes of membrane-bound and unbound (supernatant; Sup) protein extracts were analyzed by immunoblot. The integral plasma membrane protein PMCA is used as positive control. **(B)** Internalization of exogenous ApoD in BV2 is time and PQ-dependent. **(C)** After 3 h exposure to 50 nM ApoD, the internalized ApoD steadily decreases with time in BV2 cells. **(D)** Exogenously added ApoD traffics to the late-endosome-lysosomal compartment of primary mouse microglia in control conditions, but with a lower co-localization index than that of primary astrocytes. *N* = 2 independent experiments **(A)** or three independent experiments **(B—D)**. Co-localization index in **(D)** measured in *z*-stacks from 12 microglial cells and 8 astrocytes. Asterisks point to significant differences. Calibration bars **(B)**: 20 μm; **(C)**: 50 μm; **(D)**: 10 μm.

As previously described for neuronal cells (Corraliza-Gomez et al., 2022), membrane preparations from BV2 cells were exposed *in vitro* to purified human ApoD. These experiments demonstrate a dose-dependent partitioning of ApoD with microglial membranes (Figure 6A), a necessary interaction for the internalization demonstrated above (Figure 2C).

Apolipoprotein D internalization is promoted by pro-oxidant stimuli in astrocytes and neuronal cells, a process evidenced in the first 2 h of exposure to ApoD in these cell types (Pascua-Maestro et al., 2017). In microglial cells, however, longer times are required to detect an increased internalization of ApoD (Figure 6B).

Exogenously-added native and glycosylated ApoD, as opposed to bacterial recombinant ApoD, was demonstrated to persist in astrocytes for over 48–72 h (Pascua-Maestro et al., 2017; García-Mateo et al., 2018). This persistence is not observed in microglia (Figure 6C), where a fast decay of intracellular ApoD is observed after a pulse of 24 h exposure.

Finally, we measured the co-localization of ApoD with the late endosome-lysosome marker Lamp2 and found that, as reported for astrocytes and neurons, ApoD enters this compartment in primary microglia (Figure 6D). However, this co-localization was significantly smaller than that quantified in astrocytes.

Taken together, these data reveal the importance of understanding the dynamics of ApoD intracellular traffic to properly interpret its impact on microglial physiology. They strongly suggest that ApoD modulates the microglial responses and functions described above through a different mechanism, probably most related to modulation of signaling cascades, which do not rely on its stable presence and maintenance role in the endolysosomal compartment.

## Discussion

A wealth of scientific data supports that the Lipocalin ApoD has a role in glial reactivity upon nervous system tissue damage, and experimental confirmation of these responses by astrocytes and myelinating glia have been demonstrated in model organisms or cell culture systems (reviewed by Sanchez and Ganfornina, 2021). However, the role of ApoD on microglial function has not been explored in detail. Several reports point to an ApoD-dependent microglial reaction in response to inflammatory or neurodegenerative conditions (Do Carmo et al., 2008; Li et al., 2015), and ApoD has been found co-localizing with microglial cells in neurodegenerative conditions (Desai et al., 2005; Pascua-Maestro et al., 2020). Recent advances in decoding the molecular crosstalk between microglia and astrocytes are of paramount importance to understand glial function in health and disease (Liddelow et al., 2020; Garland et al., 2022). Therefore, an experimental analysis of the role of ApoD in microglia was required.

Our current work shows that a lack of ApoD results in an increased microglial density in the hippocampus of young mice (Figures 1C, D) that contrasts with the stable density observed along the mouse lifespan (Askew et al., 2017). Microglia numbers in ApoD-KO hippocampi return to normal values with aging, indicating that they undergo a transient exit from the homeostatic control of cell numbers during early adulthood. This phenomenon might alter the epigenetic imprinting of microglia and affect their future response upon aging and neurodegeneration, a phenomenon worth to pursue to further understand the factors regulating priming or immune memory processes in the brain (Fernández-Arjona et al., 2022a,b). Our *in silico* analysis (Figures 1A, B) favors this hypothesis since it reveals that ApoD expression conditions the microglia-specific transcriptome profile during the aging process.

Two well-known responses of microglia, to homeostatic/ground signals or to disturbing environmental stimuli, are the release of soluble factors such as cytokines and the acquisition of a phagocytic phenotype, either directed toward the injury cause or to the remains of damaged tissue.

To test the role of ApoD on microglial responses, we first explored the secretion of a set of classical inflammatory or anti-inflammatory cytokines from primary mouse microglia. A first observation is that under basal conditions, WT microglia secrete slight amounts of TNFα, IL6, and IL4, while IL1β and IL10 are below detection levels in our assay. The addition of PQ or Aβ oligomers, at concentrations not compromising cell survival in primary microglia (Supplementary Figure 2B), does not induce a differential secretion of the studied cytokines. This absence of cytokine response to harmful stimuli has been reported by other researchers (Klintworth et al., 2009; Kuter et al., 2022; Quiroga et al., 2022), and confirms that our cultured primary microglia are in a homeostatic/ground state. Nonetheless, they clearly respond to strong inflammatory stimuli, such as LPS (Supplementary Figure 1D), secreting inflammatory mediators in amounts similar to those reported by other authors (Shippy et al., 2022). Interestingly, a short-term pre-exposure to exogenously added ApoD, that would mimic a pulse of expression by neighboring cells in the brain, induces a significant and specific release of TNFα, IL6 and IL10, leaving IL4, and IL1β unaffected. Moreover, when exposure to ApoD is followed by PQ or Aβ oligomers, a distinctive cytokine secretion pattern is produced. These ApoD-induced responses are also present in female microglia, that show secretory patterns similar to those of male microglia with the exception of IL10 release, which is suppressed, and IL4 secretion that is significantly higher than in males. This IL4 enhanced expression by female microglia can be behind the differential neuroprotective function of IL4 reported for female mice subjected to experimental stroke (Xiong et al., 2015).

The general conclusion derived from the data obtained with WT microglial cultures is that ApoD selectively modulates the release of specific cytokines in microglia in a sex and stimulus-dependent manner, which in turn could elicit a varied downstream inflammatory response. ApoD might be considered a preconditioning agent derived from astrocytes in response to harmful stimuli, such as OS and amyloid deposition, which influences in a stimulus-specific manner the final response of microglia. The consistently decreased TNFα, IL6, and IL10 release generated by pairing an acute exposure to ApoD and Aβ oligomers is intriguing, and suggests a putative competitive effect of Aβ oligomers on the cytokine release induced by ApoD. Functional relationships like this might underlie the biological limits of microglia in the advanced Alzheimer’s brain where both, ApoD and Aβ oligomers, are elevated. However, the overall increased cytokine release when ApoD is paired with the ROS-generator PQ suggests a classical preconditioning effect for microglial response to OS, as has been previously reported for other stimuli like LPS (Mizobuchi and Soma, 2021; Kuter et al., 2022).

It is expected that ApoD would act in concert with other lipid-binding proteins in different physiological or pathological conditions. For example, other apolipoproteins (ApoE and ApoJ) participate in the microglial clearing of Aβ oligomers, and thus contribute to the protection of neural cells (Xie et al., 2005; Qin et al., 2006). Also, other members of the Lipocalin family, expressed by glia as well (Lcn2, Orm2, and C8g), have been shown to protect cells from stressful stimuli. However, they appear to exert opposite roles depending on whether they activate (Lcn2) or inhibit (Orm2 and C8g) certain microglial inflammatory responses (Jo et al., 2017; Bhusal et al., 2021; Kim et al., 2021). ApoD can now be added to this set of signals conveyed by lipid binding proteins to the highly sensitive microglia, which would integrate them to produce specific responses.

When we analyze the responses of microglia derived from ApoD-KO brains, a noticeably different functional scenario emerges. We observe a consistently dampened release of cytokines, both by male and female microglia. It is important to notice that, while WT microglia have been cultured with conditioned media from ApoD-expressing astrocytes up to 5 h before being exposed to our experimental conditions, ApoD-KO microglia have always lived in a constitutive absence of ApoD. Our results suggest the existence of a long-term instructive or memory function of ApoD that might be based on the existence of an astrocyte-microglia crosstalk in response to environmental signals in the healthy brain along its lifespan. This necessary crosstalk would then condition the future response to dangerous stimuli upon disease.

Our experiments to analyze the role of ApoD on the phagocytic activity of microglia are based on the known modulatory role of ApoD in myelin phagocytosis reported on Schwann cells, macrophages, and astrocytes, where ApoD was shown to optimize phagocytosis and promote myelin degradation (reviewed by Sanchez and Ganfornina, 2021). Our results here also uncover that BV2 microglia are not indifferent to ApoD when exposed to brain-derived myelin particles. We have confirmed with two different techniques (FACS and fluorescence microscopy) that BV2 microglia have an enhanced uptake of myelin particles isolated from ApoD-KO mouse brains. However, these results contrast with those reported for mouse primary macrophages (García-Mateo et al., 2014), which show reduced phagocytosis of brain myelin particles from ApoD-KO mice in the same time windows explored in this work. To account for this discrepancy, we must take into account that peritoneal macrophages used in our previous work had been pre-activated by thioglycolate, while BV2 microglial cells were not stimulated before being exposed to myelin, an experimental difference that might affect their phagocytic phenotype. On the other hand, the striking and consistent effect on BV2 microglia of exogenously added ApoD does parallel the decreased phagocytosis levels obtained in macrophages. Pre-exposure to ApoD, possibly activating signaling pathways through its interaction with microglial membranes, drastically reduces phagocytosis regardless of the myelin ApoD genotype. This result was also reported for ApoD-KO macrophages, using similar protein concentrations and temporal window for the phagocytosis assay (García-Mateo et al., 2014). BV2 microglia, with many subculture steps *ex vivo*, would be in this sense similar to primary ApoD-KO microglia. In any case, we must be cautious when comparing macrophage behaviors with microglia (primary cultures or cell lines), both *in vitro* and *in vivo*. Whether the results obtained with macrophages and microglia do reflect similar functional states is still under debate (Brandenburg et al., 2020).

Finally, our analysis of the degradation time course of myelin particles engulfed by naïve BV2 microglia reveals a similar rate of degradation for myelin of either genotype, also in contrast with the slower degradation of ApoD-KO myelin by thioglycolate-activated macrophages (García-Mateo et al., 2014). Our data show that the acute exposure to ApoD reduces myelin degradation rate. In summary, we describe here genotype-dependent differences in myelin uptake by microglia that might be due to the constitutive defects in myelin structure and composition caused by the long-term absence of ApoD (García-Mateo et al., 2018). Instead, pre-exposing microglial cells to soluble ApoD, as expected in a neural environment where ApoD-expressing astrocytes and/or myelinating glia react to tissue damage, reduces the uptake and degradation of myelin remains, and is expected to concur with ApoD-dependent specific patterns of cytokine secretion. We can conclude that short-term exposure to ApoD induces microglia to switch from their resting state to a secretory but less phagocytic phenotype, while long-term absence of ApoD leads to attenuated cytokine induction and increased myelin uptake, supporting a role for ApoD as priming or immune training factor.

In spite of the ApoD-dependent functional alterations of microglia, both in the mouse brain and in culture systems, we here demonstrate that mouse microglia do not express ApoD, neither under basal or OS conditions (Figures 2A, B). These data agree with the negligible transcript levels recovered for this gene from individual microglia RNA-seq experiments (Saunders et al., 2018; Hammond et al., 2019). However, as reported for other cell types, extracellular ApoD can be internalized by microglia (Figures 2C, 6B, D).

Internalization of ApoD and traffic to the endosomal-lysosomal compartment has been demonstrated to be a requirement for various ApoD functions, such as cell survival upon OS or neurodegenerative conditions, or modification of glycocalyx composition required for the completion of myelin compaction (reviewed by Sanchez and Ganfornina, 2021). Our experiments with microglial cells exposed to harmful stimuli such as PQ-derived OS (Figure 3A), however, show an apparent lack of a protective role of ApoD, contrary to the ApoD neuroprotection found in other cells and model organisms. Similarly, microglia exposed to noxious Aβ oligomers are not protected by ApoD (Figure 3B), though ApoD-KO mice show more Aβ plaques and more microglia (Li et al., 2015). These data are compatible with the known microglial failure in Alzheimer’s disease brains (Streit et al., 2020), where microglia reach their limit and cannot succeed in clearing Aβ products and instead turn to a secretory state that promotes inflammation, recruit more immune cells, and contribute to the damage.

Only limited reports describe improvement of microglial survival by soluble factors, mainly involving the glial-derived factors CSF-1/TGFβ and the TREM2 receptor (Bohlen et al., 2017; Zheng et al., 2017). Here we observe a pro-survival effect of soluble ApoD in control conditions (Figure 3), in agreement with results obtained from fibroblasts (Pascua-Maestro et al., 2020), neuronal and glial cell lines (Martínez-Pinilla et al., 2015; Pascua-Maestro et al., 2017). This limited pro-survival effect of ApoD on microglia and the effects on myelin degradation, are coherent with the finding that ApoD presence in the lysosomal compartment is transient (Figure 6C), a process that differs from the stable presence in lysosomes of other cell types and reveal the importance of analyzing the subcellular traffic of ApoD to understand its impact on microglial physiology. Also, the modulation of this traffic by OS is milder and slower in microglia compared to astrocytes or neurons. Therefore, ApoD roles in microglia are predicted to be mediated by triggering processes downstream of signaling cascades after interaction with particular liquid-ordered membrane domains (Corraliza-Gomez et al., 2022), rather than by direct effector functions on membrane managing and preservation of lysosomal pH, as it is the case for other cell types in the nervous system. The dual signaling role uncovered by this first exploration of ApoD function on microglia might be based on the modulation of other lipid raft-dwelling proteins like APP, that can influence pathogenesis (Cordy et al., 2003; Minami et al., 2011), or Toll-like receptors that regulate brain immune responses to disease (Fernández-Arjona et al., 2022b).

In summary, our results suggest that ApoD modulation of microglial responses to aging and neurodegeneration-related stimuli reveals both long-term priming or instructive roles and short-term acute effects, in inflammation-signaling molecules and phagocytic activity and efficiency, which do not depend on its permanence in the lysosomal compartment.

## Data availability statement

The original contributions presented in this study are included in this article/Supplementary material, further inquiries can be directed to the corresponding authors.

## Ethics statement

The animal study was reviewed and approved by the University of Valladolid Animal Care and Use Committee.

## Author contributions

MC-G, DS, and MG contributed to conception and design of the study. MC-G, BB, DS-C, MV, MP, JM-D, DS, and MG designed and performed the experiments. MC-G, JV, DS, and MG analyzed the data. DS and MG wrote the first draft of the manuscript. MC-G and MG reviewed the manuscript. All authors contributed to manuscript revision, read, and approved the submitted version.
