# Supplementary material for: Dual role of Apolipoprotein D as long-term instructive factor and acute signal conditioning microglial secretory and phagocytic responses
Source: Front Cell Neurosci. 2023 Jan 26;17:1112930. doi: 10.3389/fncel.2023.1112930 (PMC9908747; doi:10.3389/fncel.2023.1112930)
Supplement: Supplementary file 2 [file Data_Sheet_2.docx]

Supplementary Files and Figures

**Dual role of Apolipoprotein D as long-term instructive factor and acute signal conditioning microglial secretory and phagocytic responses.**

**Miriam Corraliza-Gomez**^1^**, Beatriz Bendito**^1^**, David Sandonis-Camarero**^1^**, Jorge Mondejar**^1^**, Miguel Villa**^1^**, Marta Poncela**^1^**, Jorge Valero**^2^**, Diego Sanchez**^1^**^#^* and Maria D. Ganfornina**^1^**^#^***

^1^Instituto de Biología y Genética Molecular, Unidad de Excelencia, University of Valladolid-CSIC, 47003 Valladolid, Spain.

^2^Instituto de Neurociencias de Castilla y León. University of Salamanca, 37007 Salamanca, Spain.

***Correspondence:** Maria D. Ganfornina and Diego Sanchez

e-mails: [mdganfornina@uva.es](mailto:mdganfornina@uva.es); [opabinia@ibgm.uva.es](mailto:opabinia@ibgm.uva.es) and [dsanchez@uva.es](mailto:dsanchez@uva.es)

# Supplementary File 1.

Microglial gene-enrichment bioinformatics analysis of mouse brain genes with ApoD-dependent transcriptional expression in response to physiological aging.

# Supplementary Figures, 1 to 3.


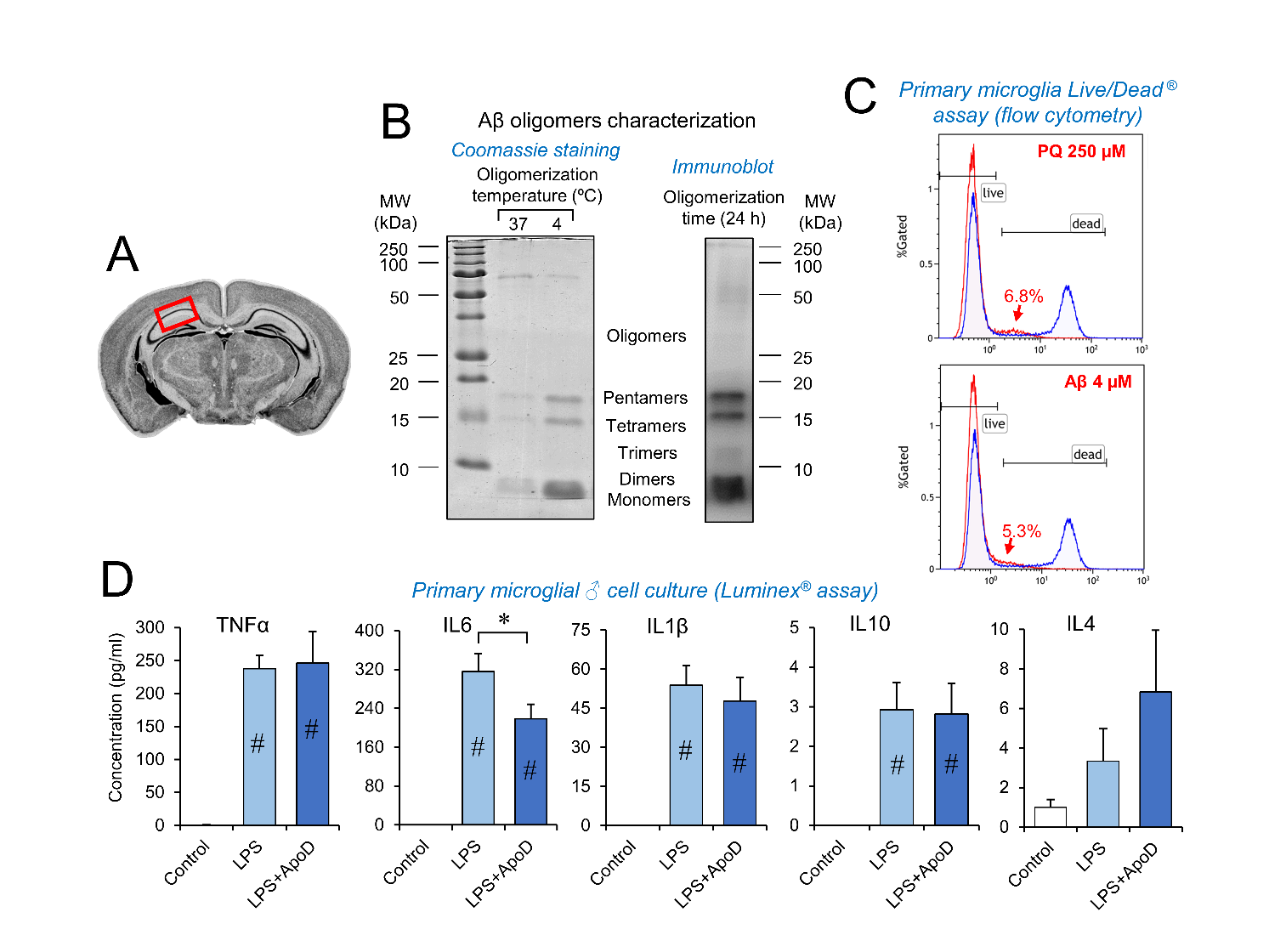


**Supplementary figure 1.** **A.** Coronal view of the region (boxed in red) sampled in the histochemical analysis. The histological section shown comes from the mouse brain library atlas (http://www.mbl.org/). **B.** Aβ oligomers characterization. Aβ oligomers were visualized either by Coomassie staining or by immunoblot. Oligomerization was performed at 37ºC or 4ºC for 24 hours. 4ºC was selected as the temperature of choice. **C.** Cell viability measured with the LIVE/DEAD Fixable Dead Cell Stain assay. Live cells react with the dye only on their surface, while cells with compromised membranes react with the dye throughout their volume, yielding a strong fluorescent signal. Control mixed population of live and dead cells is used to select the gate for dead cells (blue). Cells treated with PQ (250 µM) or Aβ oligomers (4 µM) are shown in red. A small percentage of cells die with these stimuli concentrations, which are 10 times or 4 times higher respectively than those used for cytokine secretion profiling. **D.** Cytokines secretion evaluated in WT male primary microglia upon LPS (100 ng/ml) exposure (light blue) or LPS preceded by ApoD (50 nM) addition (dark blue) as schematically represented in Fig. 4. Asterisks point to significant differences among groups of each graph, and # represent differences with respect to control conditions, evaluated by three-way ANOVA followed by Holm-Sidak pairwise comparisons.


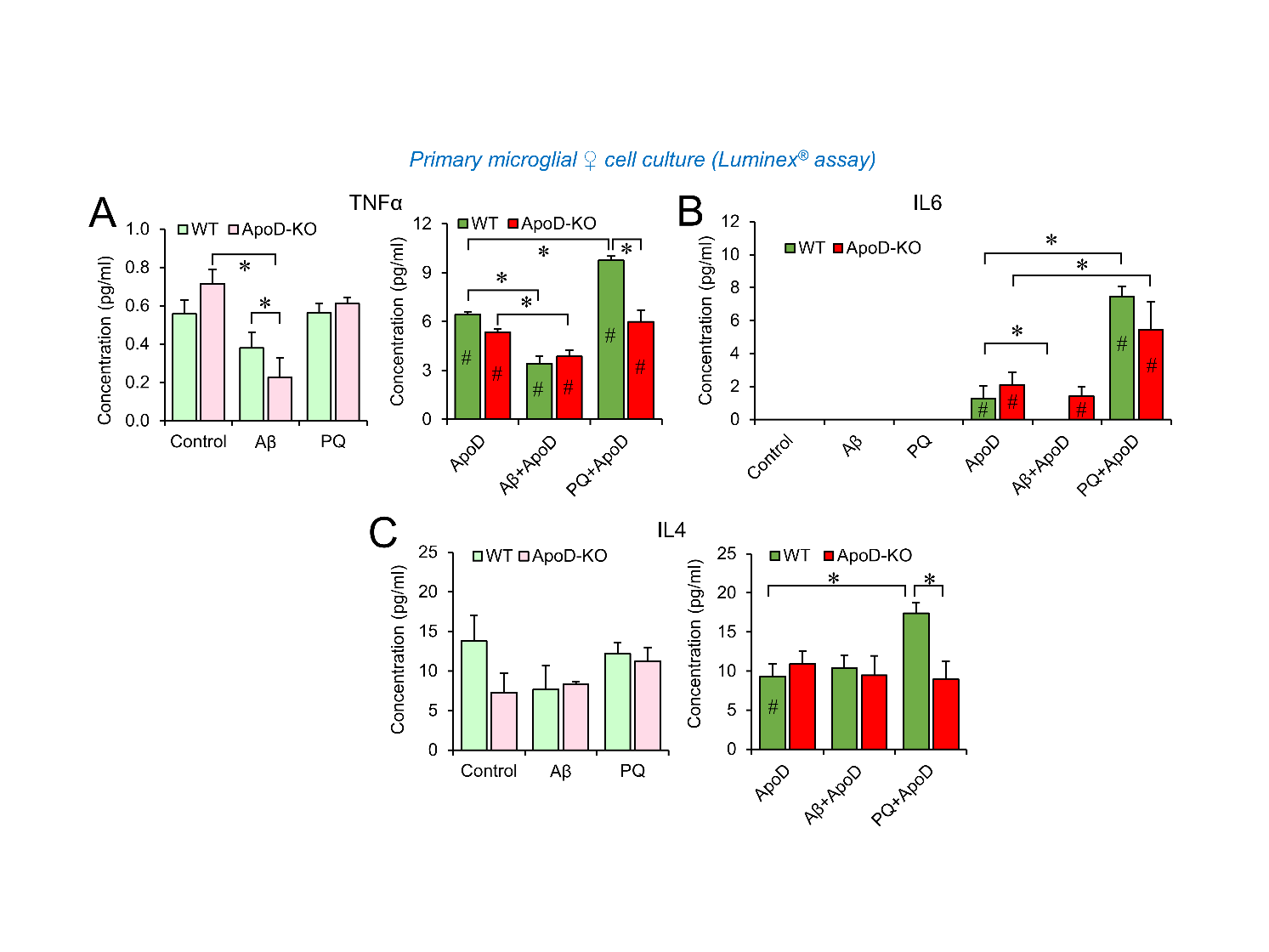


**Supplementary figure 2. Cytokine secretion profiles of female primary microglia in control conditions, PQ-induced OS or exposure to Aβ-oligomers.** Luminex multiplex assay is used with microglia culture media after 18 h incubation under different conditions (control, 1 µM Aβ oligomers, or 25 µM PQ). Light green and pink bars represent concentration of each cytokine under control conditions for primary microglia from WT (light green) or ApoD-KO mice (pink). Dark green and red represent concentration of each cytokine secreted by WT or ApoD-KO primary microglia when stimuli were preceded by the addition of purified human ApoD (50 nM) to the culture medium. ApoD is maintained during the treatment period. Each profile represents two technical replicas of two primary cultures, each obtained from two separate newborn mice per sex. Asterisks point to significant differences among groups within each graph, and # point to differences between conditions, with or without ApoD acute exposure, evaluated by ANOVA followed by Holm-Sidak pairwise comparisons.


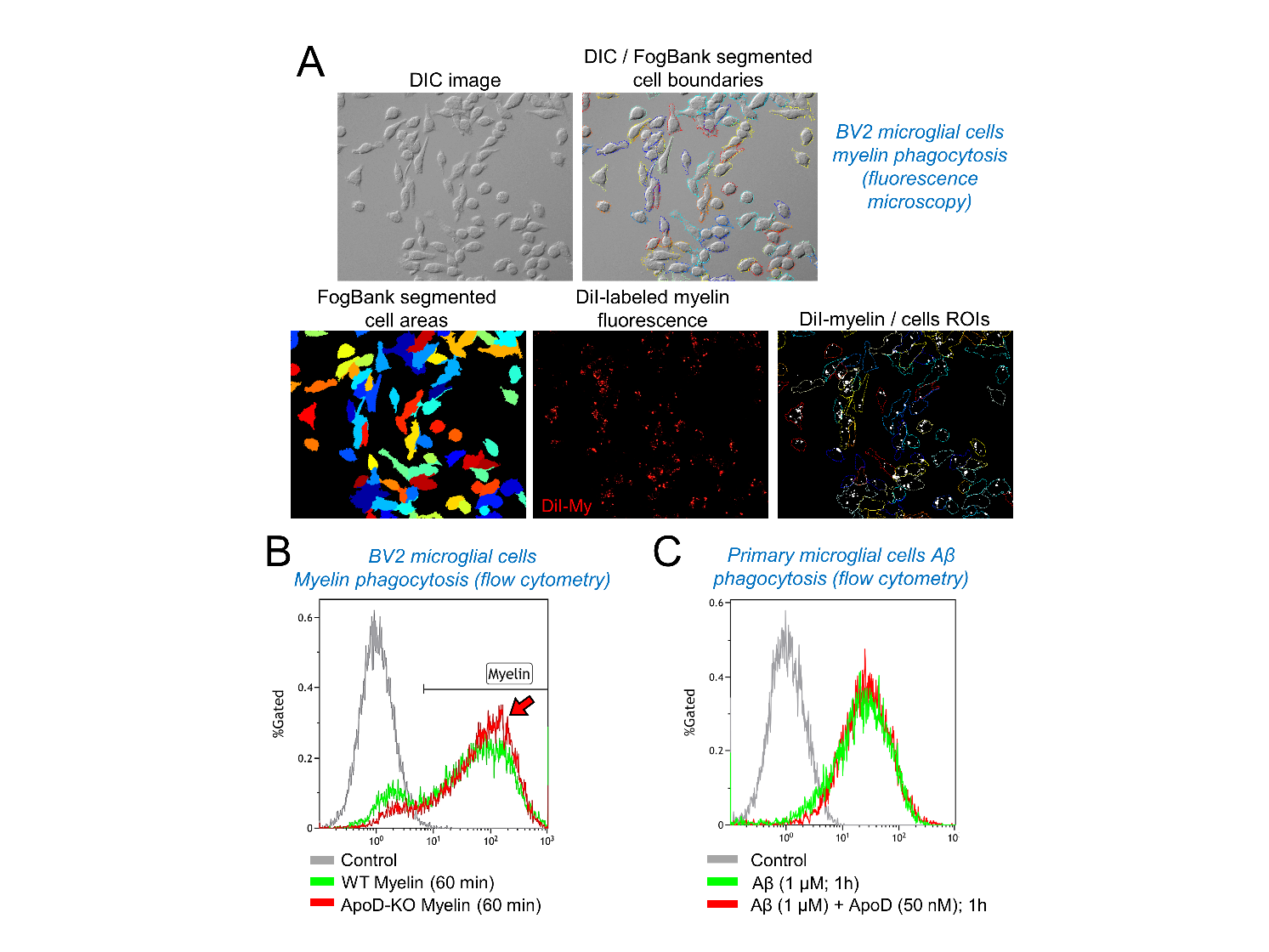


**Supplementary figure 3. A.** Representative images showing the steps followed to study DiI-labeled myelin phagocytosis in BV2 cells, using DIC and fluorescence microscopy images and the FogBank segmentation method. ROI analyses were performed with a custom-made Fiji macro, selecting only non-overlapping cells. **B.** Flow cytometry experiment demonstrating that myelin from ApoD-KO mice is phagocytosed more efficiently by BV2 cells than WT myelin. **C.** Flow cytometry experiment showing the lack of an effect of ApoD addition on the uptake of Aβ oligomers by primary microglial cells.
